# Supplementary material for: Sensitivity analysis of shock distributions in the world economy
Source: PLoS One. 2023 Oct 30;18(10):e0293524. doi: 10.1371/journal.pone.0293524 (PMC10615321; doi:10.1371/journal.pone.0293524)
Supplement: S1 Appendix — (PDF) [file pone.0293524.s001.pdf]

Appendix S1: Theoretical model

To understand the structure of the world economy we consider the model introduced in [1], here, extended for WIOD.

Model formulation: Consider a world with $J \geq 1$ countries (economies) $\{con_1, \ldots, con_J\}$ and $S \geq 1$ sectors (industries) $\{sec_1, \ldots, sec_S\}$ as a network $G = (V, E)$ of N nodes in which each node represents a country-industry pair. Country-industry pairs $(con_{\hat{i}}, sec_r)$ are mapped to the nodes in V with $(con_{\hat{i}}, sec_r) \rightarrow (\hat{i} - 1)S + r$, for $\hat{i} = 1, \ldots, J$ and $r = 1, \ldots, S$. Note that the nodes $1, \ldots, S$ correspond to the country 1, con_1, the nodes $S + 1, \ldots, 2S$ are related to the country 2, con_2, and so on.

Consider a static world-economy consisting of N competitive county-industry pairs, each producing a distinct product. Each product can be either consumed by the households or used as an intermediate input for production of other goods. Firms in each industry employ Cobb-Douglas production technologies with constant returns to scale to transform intermediate inputs and labor into final products. In particular, the output y_i of country-industry pair i is given by

$$y_i = \zeta_i \xi_i \left[\ell_i\right]^{\alpha_i} \prod_{j=1}^{N} \left[\chi_{ji}\right]^{a_{ji}}, \tag{1}$$

χ_{ji} is the amount (quantity) of good j produced by country-industry pair j used as input by country-industry pair i, ℓ_i is the amount of labor hired by firms in country-industry pair i, ζ_i is a Hicks-neutral productivity shock, and $\xi_i > 0$ is a normalization constant. The exponent $a_{ji} \geq 0$ in (1) represents the share of good j (produced by county-industry pair j) that are used in the production technology of good i (in the county-industry pair i), i.e., the ji-th entry of the matrix $\mathbf{A}$. A larger a_{ji} means that good j is more important in producing in, whereas $a_{ji} = 0$ implies that good j is not a required input for n's production technology. We assume that, for each i, $\alpha_i > 0$, and $a_{ji} \geq 0$ for all j, and

$$\alpha_i + \sum_{j=1}^{N} a_{ji} = 1, \tag{2}$$

so that the production function of each country-industry pair exhibits constant returns to scale.

As the output of each industry is used as input for other industries or consumed in the final good sector, the market-clearing condition for country-industry pair i can be written as

$$y_i = c_i + \sum_{j=1}^{N} \chi_{ij}, \tag{3}$$

where c_i is the (final) amount of goods produced in the country-industry pair i that are consumed (final consumption of the output of country-industry i). The preference side of this world economy is summarized by a representative household with a utility

function

$$u(c_1, c_2, \ldots, c_N, \ell) = \prod_{i=1}^{N} [c_i]^{\beta_i}, \tag{4}$$

where $\beta_i \in (0,1)$ designates the weight of the good i (produced by country-industry pair i) in the representative household's preferences (with the normalization $\sum_i^N \beta_i = 1$). Denoting the price of the output of country-industry n by p_n, and assuming that income comes only from labor, $\omega\ell$, the representative household's budget constraint can be written as

$$\sum_{i=1}^{N} p_i c_i = \omega\ell.$$

Model properties: We focus on the competitive equilibrium of this static economy, so that all country-sector pairs (firms) maximize profits and the representative household maximizes its utility, in both cases taking all prices as given, and the market-clearing conditions for each good and labor are satisfied. The Cobb-Douglas production functions in (1), combined with profit maximization, imply

$$a_{ji} = \frac{p_j \chi_{ji}}{p_i y_i}, \tag{5}$$

$$\alpha_i = \frac{\omega \ell_i}{p_i y_i}. \tag{6}$$

Utility maximization in turn yields

$$\frac{p_i c_i}{\beta_i} = \frac{p_j c_j}{\beta_j}. \tag{7}$$

Since total household income is equal to labor income, we have

$$\sum_{i=1}^{N} p_i c_i = \omega\ell,$$

which yields

$$p_i c_i = \beta_i \omega \ell, \tag{8}$$

for all i. We assume that (i) labor is the only primary factor of production, (ii) all firms make zero profits, and (iii) total labor supply is normalized to 1, $\ell = 1$, gross domestic product in the world economy, or gross world product (GWP), is equal to the market wage, ω:

$$\text{GWP} = \omega = \sum_i p_i c_i. \tag{9}$$

Using (3), (5), and (8) for equilibrium revenues per country-industry pair, we have

$$\begin{aligned} r_i \equiv p_i y_i &= p_i \sum_j \chi_{ij} + c_i p_i \\ &= p_i \sum_j \frac{a_{ij} p_j y_j}{p_i} + \beta_i \omega \ell \\ &= \sum_j a_{ij} r_j + \beta_i \omega. \end{aligned}$$

Writing $\mathbf{r} = [r_1, \ldots, r_N]^T$ and $\boldsymbol{\beta} = [\beta_1, \ldots, \beta_N]^T$, the last equation can be written as

$$\mathbf{r} = \omega \left(\mathbf{I} - \mathbf{A}\right)^{-1} \boldsymbol{\beta} \; . \tag{10}$$

We now define the world influence index as

$$\mathbf{v} = \left(\mathbf{I} - \mathbf{A}\right)^{-1} \boldsymbol{\beta}. \tag{11}$$

Therefore, by combining (10) and (11) with (9) we have $\mathbf{v} = \mathbf{r}/\omega$ or

$$v_i = \frac{p_i y_i}{\sum_j p_j c_j} = \frac{p_i y_i}{\text{GWP}}. \tag{12}$$

These quantities are also known as Domar weights (defined as world-sectoral sales divided by GWP).

Remarks: Several remarks are in order.

First, in a special case, when $\alpha_i = \alpha$ for all i, since $\alpha + \sum_j a_{ji} = 1$ and defining Ω_{ji} such that $a_{ji} = (1-\alpha)\Omega_{ji}$ with $\sum_j \Omega_{ji} = 1$, Eq. (11) reduces to

$$\mathbf{v} = \left[\mathbf{I} - (1-\alpha)\boldsymbol{\Omega}\right]^{-1} \; \boldsymbol{\beta} \tag{13}$$

where $\boldsymbol{\Omega} = [\Omega_{ji}]$ is $N \times N$ stochastic matrix.

Second, if country-industry pairs source equally from all other pairs and also sell in equal proportions to final demand, (13) collapses to $\mathbf{v} = 1/N$. This is a restatement of the classical diversification argument by Lucas [2]: aggregate volatility is then proportional to $1/\sqrt{N}$ and shocks to individual sectors in a country wash out in the aggregate from a law of large numbers argument.

Third, with homogeneous input shares and heterogeneity in final demand, (13) collapses to

$$\mathbf{v} = \left[\mathbf{I} - \frac{(1-\alpha)}{N}\right]^{-1} \; \boldsymbol{\beta}.$$

For large N, this can be approximated as $\mathbf{v} \approx \boldsymbol{\beta}$. Hence the influence of individual firms is proportional to their sales to final demand. Moreover, if $\alpha = 1$, (13) becomes $\mathbf{v} = \boldsymbol{\beta} = \frac{\mathbf{r}}{\mathbf{r1}}$. This is a direct statement of the granular hypothesis presented by Gabaix [3]: in the presence of a sufficiently skewed sales Herfindahl index that is consistent with a power law with fat tails, shocks to large firms can contribute significantly to aggregate fluctuations.

Finally, with heterogeneous input shares and homogeneous sales to final demand, that is $\beta_i = 1/N$, (13) collapses to

$$\mathbf{v} = \frac{1}{N}[\mathbf{I} - (1-\alpha)\boldsymbol{\Omega}]^{-1}\mathbf{1}.$$

This is a modified influence vector introduced in [1] (as the factor α does not appear in the equation): if the distribution of $\mathbf{v}$ is consistent with a power law distribution with fat tails, idiosyncratic shocks to important suppliers can propagate through the network of production and show up in the aggregate of the economy.

Note on GWP: Plugging χ_{ji} and ℓ_i from (5) and (6) into i's production function (1), and setting (which simplifies our key expressions without any bearing on our results)

$$\xi_i \alpha_i^{\alpha_i} \prod_j [a_{ji}]^{a_{ji}} = 1,$$

we have

$$\begin{aligned} y_i &= \zeta_i \xi_i \left[\frac{\alpha_i p_i y_i}{\omega}\right]^{\alpha_i} \prod_j \left[\frac{a_{ji} p_i y_i}{p_j}\right]^{a_{ji}} \\ &= \zeta_i \xi_i \alpha_i^{\alpha_i} \frac{1}{\omega^{\alpha_i}} p_i y_i \prod_j [a_{ji}]^{a_{ji}} \prod_j [p_j]^{-a_{ji}} \\ &= \zeta_i \left[\frac{p_i y_i}{\omega}\right]^{\alpha_i} \prod_j \left[\frac{p_i y_i}{p_j}\right]^{a_{ji}}. \end{aligned}$$

Therefore,

$$\alpha_i \log \omega = \varepsilon_i + \log p_i - \sum_j a_{ji} \log p_j,$$

where $\varepsilon_i = \log \zeta_i$ are microeconomic shocks that are i.i.d. across country-industry pairs, are symmetrically distributed around the origin with full support over $\mathbb{R}$, and have a finite standard deviation, which we normalize to one. The previous equation implies that

$$\log \frac{p_i}{\omega} = \sum_j a_{ji} \log \frac{p_j}{\omega} - \varepsilon_i.$$

Rewriting this system of equations in matrix form we get that

$$\hat{\mathcal{P}} = \mathbf{A}^T \hat{\mathcal{P}} - \varepsilon,$$

where

$$\begin{aligned} \varepsilon &= (\varepsilon_1, \ldots, \varepsilon_N)^T \\ \hat{\mathcal{P}} &= (\log(p_1/\omega), \ldots, \log(p_N/\omega))^T \end{aligned}$$

denote the vectors of log relative prices and productivity shocks, respectively. Consequently, the equilibrium vector of (log) relative prices is given by

$$\hat{\mathcal{P}} = -(\mathbf{I} - \mathbf{A}^T)^{-1} \varepsilon = -\mathbf{L}\varepsilon, \tag{14}$$

where $\mathbf{L} = [l_{ij}]$ is the Leontief inverse. This can be also written as

$$\log \frac{p_i}{GWP} = -\sum_{j=1}^{N} l_{ji} \varepsilon_j. \tag{15}$$

Equation (11) implies $v_i = p_i y_i / GWP = \sum_{j=1}^{N} l_{ij} \beta_j$. Combining with equation (15), the following result holds:

$$\log(y_i) = \sum_{j=1}^{N} l_{ij} \varepsilon_j + \delta_i, \tag{16}$$

where δ_i is some constant that is independent of the shocks. Multiplying both sides of (15) by β_i and summing over all country-industry pairs i lead to

$$\log(GWP) = \sum_{j=1}^{N} \sum_{i=1}^{N} \beta_i l_{ji} \varepsilon_j + \sum_{i=1}^{N} \beta_i \log p_i.$$

By normalizing the price index $\prod_i p_i^{\beta_i} = 1$, we obtain

$$\log(\text{GWP}) = \sum_i \beta_i \sum_j l_{ji} \varepsilon_j \tag{17}$$

Since $\mathbf{L} = \widetilde{\mathbf{L}}^T$, from (17) and (11) it follows the aggregate output of the world economy is given by

$$y \equiv \log(\text{GWP}) = \sum_{i=1}^{N} v_i \varepsilon_i. \tag{18}$$

Equation (18), related to [1, 3, 4], shows that in a competitive world economy with constant returns to scale technologies, world aggregate output is a linear combination of country-industry level productivity shocks, with coefficients v_n given by Domar weights. Moreover, the Domar weight of each country-industry pair n depends only on the preference shares, $\beta_1, \ldots, \beta_N$, and the corresponding column of the world-economy's Leontief inverse.

Empirical example: Let us return to our aggregated economy example and calculate the corresponding Domar weights $\mathbf{v}$, the labor input share α and the preference shares β. The first quantity can be directly calculated using equation (11). Moreover, using equation (6) and the properties of the World input output table we can estimate the entries of the α vector as

$$\alpha_i = \frac{w_i}{x_i}.$$

In a similar fashion, due to the properties described with (7) we can estimate the elements of the β vector as

$$\beta_n = \frac{f_i}{\sum_j f_j}.$$

References

1. Acemoglu D, Carvalho VM, Ozdaglar A, Tahbaz-Salehi A. The network origins of aggregate fluctuations. Econometrica. 2012 Sep;80(5):1977-2016.
2. Robert Lucas J. Understanding Business Cycle. InStabilization of the Domestic and International Economy: Carnegie-Rochester Conference Series on Public Policy 1977.
3. Gabaix X. The granular origins of aggregate fluctuations. Econometrica. 2011 May;79(3):733-72.
4. Hulten CR. Growth accounting with intermediate inputs. The Review of Economic Studies. 1978 Oct 1;45(3):511-8.
