# Supplementary material for: Sensitivity analysis of shock distributions in the world economy
Source: PLoS One. 2023 Oct 30;18(10):e0293524. doi: 10.1371/journal.pone.0293524 (PMC10615321; doi:10.1371/journal.pone.0293524)
Supplement: S2 Appendix — (PDF) [file pone.0293524.s002.pdf]

Appendix S2: Proof of the theorems

In the defining matrix derivatives we follow references [1–3]. Let $\mathbf{A}$ be a matrix; the (i, j) entry of the matrix $\mathbf{A}$ is a_{ij} and we write $\mathbf{a}(i, :)$ for the i-th row of $\mathbf{A}$, and $\mathbf{a}(:, j)$ for the j-th column of $\mathbf{A}$, For two matrices $\mathbf{A}$ and $\mathbf{B}$, the Hadamard, or elementwise product is $\mathbf{A} \odot \mathbf{B} = [a_{ij} b_{ij}]$ and the Kronecker product is $\mathbf{A} \otimes \mathbf{B} = [a_{ij} \mathbf{B}]$. The vec operator transforms a $m \times n$ matrix $\mathbf{A}$ into a mn-dimensional vector:

$$\operatorname{vec} \mathbf{A} = \begin{bmatrix} \mathbf{a}(:, 1) \\ \vdots \\ \mathbf{a}(:, n) \end{bmatrix}$$

Let $\mathbf{y}$ be an $n \times 1$ vector and $\mathbf{x}$ be a $m \times 1$ vector. The derivative of $\mathbf{y}$ with respect to $\mathbf{x}$ is defined to be the $n \times m$ matrix whose (i, j) entry is derivative of y_i with respect to x_j, i.e.,

$$\frac{d\mathbf{y}}{d\mathbf{x}^T} = \left[\frac{dy_i}{dx_j} \right]$$

The derivative of the $m \times n$ matrix $\mathbf{Y}$ with respect to the $p \times q$ matrix $\mathbf{X}$ is the $mn \times pq$ matrix

$$\frac{d \operatorname{vec} \mathbf{Y}}{d \left(\operatorname{vec} \mathbf{X} \right)^T}$$

Let $\boldsymbol{\theta}$ be a vector of parameters (of dimension $p \times 1$) on which the entries of the matrix $\mathbf{A}$ depend. Let

$$\mathbf{L} = (\mathbf{I} - \mathbf{A})^{-1} \tag{1}$$

be the fundamental matrix of the Markov chain. The sensitivity of the fundamental matrix $\mathbf{L}$ with respect to $\boldsymbol{\theta}$ is given by

$$\frac{d \operatorname{vec} \mathbf{L}}{d \boldsymbol{\theta}^T} = \left(\mathbf{L}^T \otimes \mathbf{L} \right) \frac{d \operatorname{vec} \mathbf{A}}{d \boldsymbol{\theta}^T} \tag{2}$$

Assume now that we consider an (input/output) chain given by the matrix $\mathbf{A}$, Eq. 1. Let the (i, j) entry of the matrix $\mathbf{A}$, for fixed values of i and j, is θ and we ask the question what is the rate of change of $\mathbf{L}$ in response to a change in the the parameter θ, holding the all other entries of matrix $\mathbf{A}$ constant. Then the vector $d \operatorname{vec} \mathbf{A} / d\theta$ has only one nonzero entry at $n(i-1) + j$ which is equal to 1. The $n(i-1) + j$ column of the matrix $\left(\mathbf{L}^T \otimes \mathbf{L} \right)$ reads

$$[\ell_{j1}\ell_{1i} \quad \ell_{j2}\ell_{1i} \quad \ldots \; \ell_{jn}\ell_{1i} \; \ldots \; \ell_{j1}\ell_{2i} \quad \ell_{j2}\ell_{2i} \quad \ldots \; \ell_{jn}\ell_{2i} \; \ldots \; \ell_{j1}\ell_{ni} \quad \ell_{j2}\ell_{ni} \quad \ldots \; \ell_{jn}\ell_{ni}]^T$$

Rewriting Eq. 2 as a $n \times n$ matrix, we finally derive that the sensitivity matrix of $\mathbf{L}$ with respect to the (i, j) entry of the matrix $\mathbf{Q}$, $\mathbf{L}_{ij}^{sen}$, is

$$\mathbf{L}_{ij}^{sen} = \begin{bmatrix} \ell_{j1}\ell_{1i} & \ell_{j1}\ell_{2i} & \ldots & \ell_{j1}\ell_{ni} \\ \ell_{j2}\ell_{1i} & \ell_{j2}\ell_{2i} & \ldots & \ell_{j2}\ell_{ni} \\ \vdots & \vdots & \ddots & \vdots \\ \ell_{jn}\ell_{1i} & \ell_{jn}\ell_{2i} & \ldots & \ell_{jn}\ell_{ni} \end{bmatrix} \tag{3}$$

If the outcomes and responses are measured on different scales, it is useful to calculate elasticity measuring proportional effect of proportional perturbation. Thus, the elasticity of the fundamental matrix $\mathbf{L}$ with respect to $\boldsymbol{\theta}$ is given by

$$\mathbf{D}\left(\operatorname{vec}\mathbf{L}\right)^{-1}\left(\mathbf{L}^T\otimes\mathbf{L}\right)\frac{d\operatorname{vec}\mathbf{Q}}{d\boldsymbol{\theta}^T}\mathbf{D}\left(\boldsymbol{\theta}\right) \tag{4}$$

where $\mathbf{D}(\mathbf{a})$ of the vector $\mathbf{a}$ is a diagonal matrix whose (i,i) entry is a_i. Since, in our case, the $(i+nj-n, i+nj-n)$ entry of the diagonal matrix $\mathbf{D}\left(\operatorname{vec}\mathbf{L}\right)^{-1}$ is ℓ_{ij}^{-1}, the final result after multiplying all factors in Eq 4 is a vector, which can be rewritten as

$$\mathbf{E}(q_{ij}) = \begin{bmatrix} \ell_{j1}\ell_{i1}\frac{q_{ij}}{\ell_{11}} & \ell_{j1}\ell_{i2}\frac{q_{ij}}{\ell_{12}} & \dots & \ell_{j1}\ell_{in}\frac{q_{ij}}{\ell_{1n}} \\ \ell_{j2}\ell_{i1}\frac{q_{ij}}{\ell_{21}} & \ell_{j2}\ell_{i2}\frac{q_{ij}}{\ell_{22}} & \dots & \ell_{j2}\ell_{in}\frac{q_{ij}}{\ell_{2n}} \\ \vdots & \vdots & \ddots & \vdots \\ \ell_{jn}\ell_{i1}\frac{q_{ij}}{\ell_{n1}} & \ell_{jn}\ell_{i2}\frac{q_{ij}}{\ell_{n2}} & \dots & \ell_{jn}\ell_{in}\frac{q_{ij}}{\ell_{nn}} \end{bmatrix} \tag{5}$$

This completes the proof.

References

1. Magnus JR, Neudecker H. Matrix differential calculus with applications to simple, Hadamard, and Kronecker products. Journal of Mathematical Psychology. 1985 Dec 1;29(4):474-92.

2. Magnus JR, Neudecker H. Matrix differential calculus with applications in statistics and econometrics. John Wiley & Sons; 2019 Mar 18.

3. Caswell H. Sensitivity analysis: matrix methods in demography and ecology. Springer Nature; 2019.
